# Supplementary material for: Content-rich biological network constructed by mining PubMed abstracts
Source: BMC Bioinformatics. 2004 Oct 8;5:147. doi: 10.1186/1471-2105-5-147 (PMC528731; doi:10.1186/1471-2105-5-147)
Supplement: Additional File 2 — The original results of the above study (non-essential files are deleted to keep the file size under the limit set by BMC bioinformatics). [file 1471-2105-5-147-S2.bz2 › chilibotAdditionalFile2/dip40/101ID7937747E430/html/BCL2L1_BCL2.html]

 


 **BCL2L1** and **BCL2** 
  
Found 1846 abstracts in PubMed, retrieved 40.  
 

 What does Google say? 
 PDF only 
| .edu only 

---

**Interactive relationship** (e.g. stimulation, inhibition, etc)

**Inhibitory relationship**- Bcl xL  [ **BCL2L1** ]  is an antiapoptotic member of the Bcl 2  [ **BCL2** ]  family, which inhibits apoptosis initiated by various cellular stresses, and has a pivotal role in the survival of tumor cells.  Ref: 12810626 Cancer Res, 2003
- In addition, detailed knowledge of the structures of Bcl 2  [ **BCL2** ]  and Bcl X  [ **BCL2L1** ]  L, coupled with high throughput and computer assisted screening of chemical libraries, has led to the identification of a number of short peptides and small organic molecules capable of inhibiting Bcl 2  [ **BCL2** ]  and Bcl X  [ **BCL2L1** ]  L function.  Ref: 12886234 Leukemia, 2003
**Neutral relationship**- Bcl X  [ **BCL2L1** ]  L is a pro survival member of the Bcl 2  [ **BCL2** ]  family that plays an important role in apoptosis regulation.  Ref: 12831534 Hybrid Hybridomics, 2003
- Apoptosis was associated with the down regulation of bcl 2  [ **BCL2** ]  family members bcl x  [ **BCL2L1** ]  L and bfl A1, and the activation of caspase 3, that mediates bcl 2  [ **BCL2** ]  cleavage, resulting in the release of cytochrome c from the mitochondria.  Ref: 12816986 J Immunol, 2003
- gossypol induced apoptosis triggers down regulation of anti apoptosis Bcl 2  [ **BCL2** ]  members Bcl X  [ **BCL2L1** ]  L, Bag 1 and Mcl 1, up regulation of pro apoptosis Bcl 2  [ **BCL2** ]  member Bak, activation of caspase,, and 9, up regulation of Apaf 1, release of cytochrome c cyto c from mitochondria, and activation of both DFF45 and PARP.  Ref: 12818369 Biochem Pharmacol, 2003
- Multiplex RT PCR of apoptosis pathway related genes revealed that Bcl 2  [ **BCL2** ]  is absent and Bcl xS  [ **BCL2L1** ]  message increases in FHIT transfected clones.  Ref: 12890991 Pancreatology, 2003

**Non-interactive relationship** (e.g. studied together, co-existance, homology, etc.)

- Heme oxygenase 1, Bcl 2  [ **BCL2** ]  and Bcl xl  [ **BCL2L1** ]  were not increased in ABO incompatible grafts at any time point.  Ref: 12859529 Am J Transplant, 2003
- Curcumin causes dose dependent apoptosis and DNA fragmentation of Caki cells, which is preceded by the sequential dephosphorylation of Akt, down regulation of the anti apoptotic Bcl 2  [ **BCL2** ] , Bcl XL  [ **BCL2L1** ] , and IAP proteins, release of cytochrome c, and activation of caspase 3.  Ref: 12807727 Carcinogenesis< MedlineTA>Carcinogenesis, 2003
- In the present study, we have examined the roles played by the Bcl 2  [ **BCL2** ]  family members Bcl xL  [ **BCL2L1** ]  and Bid in regulating this response.  Ref: 12893848 J Pharmacol Exp TherJ Pharmacol Exp Ther, 2003
- Bax and Bcl xL  [ **BCL2L1** ]  proteins were not changed, although Bcl 2  [ **BCL2** ]  proteins increased with troglitazone.  Ref: 12840598 Am J Nephrol< MedlineTA>Am J Nephrol, 2003
- Bcl X  [ **BCL2L1** ]  and Bcl 2  [ **BCL2** ]  are co expressed in 89% of NPCs whereas their expression is mutually exclusive in other head and neck carcinomas particularly squamous cell carcinomas, SCC.  Ref: 12893071 Cancer Detect Prev, 2003
- To determine the molecular order among these events, we introduced inhibitors of LMP bafilomycin A 1, MMP Bcl X  [ **BCL2L1** ]  L, wild type Bcl 2  [ **BCL2** ] , mitochondrion targeted Bcl 2  [ **BCL2** ] , or viral mitochondrial inhibitor of apoptosis from cytomegalovirus, and caspases Z VAD.fmk into the system.  Ref: 12813466 Oncogene, 2003
- Treatment of IL 2Ralpha expressing cells with a specific Janus kinase 3 Jak3 inhibitor decreased expression of cyclin A, cyclin D1, Bcl X  [ **BCL2L1** ]  L, and Bcl 2  [ **BCL2** ]  proteins.  Ref: 12858347 J Cell Biochem, 2003
- The defective apoptosis seen in human cancers often results from overexpression of antiapoptotic proteins in the Bcl 2  [ **BCL2** ]  protein family, particularly Bcl 2  [ **BCL2** ]  and Bcl X  [ **BCL2L1** ]  L.  Ref: 12886234 Leukemia, 2003
- The protein expressions of bcl 2  [ **BCL2** ]  and bcl X  [ **BCL2L1** ]  L in A2780 DDP cells were 1.99 0.11 and 1.69 0.16, and significantly higher than those in A2780 cells P <.  Ref: 12816691 Zhonghua Fu Chan Ke Za Zhi, 2003
- RESULTS The mRNA expressions of bcl 2  [ **BCL2** ]  and bcl X  [ **BCL2L1** ]  L in A2780 DDP cells were 1.87 0.25 and 1.73 0.15, and significantly higher than those in A2780 cells P <.  Ref: 12816691 Zhonghua Fu Chan Ke Za Zhi, 2003
- Survivin expression did not correlate with bcl 2  [ **BCL2** ] , bcl x  [ **BCL2L1** ] , or bax expression, stage, or overall or disease free survival.  Ref: 12808063 Mod Pathol, 2003
- Antiapoptotic proteins of the Bcl 2  [ **BCL2** ]  family such as Bcl 2  [ **BCL2** ]  and Bcl x  [ **BCL2L1** ]  L are also expressed but, in contrast to c FLIP, c IAP, and X chromosome linked IAP, do not appear to be tightly regulated by the presence of the parasite.  Ref: 12874209 J Immunol, 2003
- We assessed survivin expression in ovarian carcinoma, correlating results with expression of other anti apoptotic bcl 2  [ **BCL2** ] , bcl x  [ **BCL2L1** ] , mutant p53 and pro apoptotic bax markers, with prognostic parameters, and prognosis.  Ref: 12808063 Mod Pathol, 2003
- METHODS The expressions of apoptosis associated genes bcl 2  [ **BCL2** ] , bax, bcl X  [ **BCL2L1** ]  L and bcl X  [ **BCL2L1** ]  S and the activity of caspase 3 were studied by reverse transcription polymerase chain reaction RT PCR and western blot in the cisplatin resistant A2780 DDP, COC1 DDP and sensitive human ovarian cancer cells A2780 and COC1.  Ref: 12816691 Zhonghua Fu Chan Ke Za Zhi, 2003
- Similar BCL X  [ **BCL2L1** ]  but different BCL 2  [ **BCL2** ]  levels in the two age groups of north African nasopharyngeal carcinomas.  Ref: 12893071 Cancer Detect Prev, 2003
- RESULTS The results of immunohistochemical staining revealed that expression of RelA, Bcl 2  [ **BCL2** ] , Bcl x  [ **BCL2L1** ]  L, and Ki 67 labeling index LI significantly increased in the transition from adenoma with low dysplasia to adenocarcinoma.  Ref: 12837981 Oncology, 2003
- The expression of RelA correlated inversely with the AI and correlated positively with the expression of Bcl 2  [ **BCL2** ] , Bcl x  [ **BCL2L1** ]  L, and Ki 67 LI in the transition from low grade dysplasia to adenocarcinoma.  Ref: 12837981 Oncology, 2003
- Remarkably, these events occurred in the absence of any reduction in the expression of the Bcl 2  [ **BCL2** ]  family members Bcl 2  [ **BCL2** ] , Mcl 1 and Bcl XL  [ **BCL2L1** ] , or any change in the proapoptotic molecules Bad or Bax.  Ref: 12842827 Am J Physiol Gastrointest Liver Physiol< MedlineTA>Am J Physiol Gastrointest Liver Physiol, 2003
- Selegiline and its metabolite, desmethylselegiline, reduce apoptosis by altering the expression of a number of genes, for instance, superoxide dismutase, Bcl 2  [ **BCL2** ] , Bcl xl  [ **BCL2L1** ] , NOS, c Jun, and nicotinamide adenine nucleotide dehydrogenase.  Ref: 12880486 Antioxid Redox Signal, 2003
- In these conditions, microscopic analysis revealed chromatin condensation in dying cells and the Bcl 2  [ **BCL2** ] , Bcl x  [ **BCL2L1** ]  L Bax mRNA balance was altered.  Ref: 12832039 FEBS Lett, 2003
- CD44v7 deficiency was characterized by an increase in the percentage of apoptotic cells after stimulation, increased numbers of CD95L and CD152 positive cells, low levels of the anti apoptotic proteins Bcl 2  [ **BCL2** ]  and Bcl Xl  [ **BCL2L1** ] , and decreased phosphorylation of the pro apoptotic protein BAD.  Ref: 12832452 J Leukoc Biol, 2003
- ... levels of other members of the Bcl 2  [ **BCL2** ]  family, such as Bax and Bcl x  [ **BCL2L1** ] , did not change in paclitaxel resistant cell lines.  Ref: 12815160 Mol Pharmacol< MedlineTA>Mol Pharmacol, 2003
- Accompanying the drug resistance, high levels of anti apoptotic Bcl X  [ **BCL2L1** ]  L and Bcl 2  [ **BCL2** ]  proteins were found in the mitochondria containing fraction of IL 2Ralpha expressing tumor cells.  Ref: 12858347 J Cell Biochem, 2003
- HSpin1 bound to Bcl 2  [ **BCL2** ]  and apoptosis regulator Bcl X  [ **BCL2L1** ]  Bcl x  [ **BCL2L1** ]  L, but not to proapoptotic members such as Bcl 2  [ **BCL2** ]  associated X protein and Bcl 2  [ **BCL2** ]  homologous antagonist killer, in cells treated with TNF alpha.  Ref: 12815463 Cell Death Differ, 2003
- These newly described agents hold considerable promise for enhancing the chemo and radiation sensitivities of Bcl 2  [ **BCL2** ]  and Bcl X  [ **BCL2L1** ]  L overexpressing cancers.  Ref: 12886234 Leukemia, 2003
- Unlike Bcl 2  [ **BCL2** ] , Bak stimulates several apoptosis pathways, and by interacting with Bcl 2  [ **BCL2** ]  or Bcl XL  [ **BCL2L1** ] , it counteracts the anti cell death effect.  Ref: 12836337 Nucleic Acids Res Suppl, 2001
- HSpin1, a transmembrane protein interacting with Bcl 2  [ **BCL2** ]  Bcl x  [ **BCL2L1** ]  L, induces a caspase independent autophagic cell death.  Ref: 12815463 Cell Death Differ, 2003
- CD40 mediated rescue occurs upstream of CD40 induced expression of Bcl 2  [ **BCL2** ]  and increased expression of Bcl xL  [ **BCL2L1** ] .  Ref: 12851674 Int J Oncol, 2003
- Furthermore, the association of RelA expression with tumor cell apoptosis, proliferation, and expression of Bcl 2  [ **BCL2** ]  Bcl x  [ **BCL2L1** ]  L was also studied.  Ref: 12837981 Oncology, 2003
- Here, we found that HDB induced apoptotic cell death was accompanied by upregulation of cyclin D3, Bax, and p21 and down regulation of Bcl X  [ **BCL2L1** ]  L, while HDB had no effect on the levels of Bcl 2  [ **BCL2** ]  and Bad protein.  Ref: 12822933 J Agric Food Chem< MedlineTA>J Agric Food Chem, 2003
- The expressions of bcl 2  [ **BCL2** ]  and bcl X  [ **BCL2L1** ]  L in COC1 DDP cells were also significantly higher than those in COC1 cells P <.  Ref: 12816691 Zhonghua Fu Chan Ke Za Zhi, 2003
- AIMS To correlate the expression of a series of apoptotic and oncogene markers including p53, Bcl 2  [ **BCL2** ] , BAX, Bcl XL  [ **BCL2L1** ] , p21WAF, 1 CIP1, cyclin D1, HER 2 neu in thymic epithelial tumours with histological type, stage and resectability and to determine whether the information on HER 2 neu would be valuable in identifying patients who are eligible for anti HER 2 neu treatment.  Ref: 12877732 Histopathology, 2003
- As Bcl 2  [ **BCL2** ]  and Bcl X  [ **BCL2L1** ]  are co expressed in EBV transformed B lymphocytes, we attempted to determine their status in malignant NPC cells.  Ref: 12893071 Cancer Detect Prev, 2003
- METHODS Paraffin sections were stained with monoclonal antibodies directed against RelA, Bcl 2  [ **BCL2** ] , Bcl x  [ **BCL2L1** ]  L, and Ki 67 to assess protein expression patterns in normal, adenomatous and colon cancer tissue.  Ref: 12837981 Oncology, 2003
- Moreover, Bcl 2  [ **BCL2** ]  and Bcl xL  [ **BCL2L1** ]  protect neurons from apoptosis, while Bax and Bcl xS  [ **BCL2L1** ]  may act as proapototic proteins.  Ref: 12820987 Neurochem Int, 2003
- Furthermore, Bcl 2  [ **BCL2** ]  family members Bad, Bak, and Bcl xS  [ **BCL2L1** ]  protein levels were increased in FHIT transfected clones when compared with Panc 1 cells.  Ref: 12890991 Pancreatology, 2003
- METHODS We performed Northern blot analysis for bax, bcl x  [ **BCL2L1** ]  L and bcl 2  [ **BCL2** ]  at both temperatures.  Ref: 12824868 Eur J Endocrinol, 2003
- Here we show that codelivery of DNA encoding inhibitors of apoptosis BCL xL  [ **BCL2L1** ] , BCL 2  [ **BCL2** ] , XIAP, dominant negative caspase 9, or dominant negative caspase 8 with DNA encoding model antigens prolongs the survival of transduced DCs.  Ref: 12840065 J Clin Invest, 2003
- ... other Bcl 2  [ **BCL2** ]  family proteins including Bax, Bcl 2  [ **BCL2** ] , Bcl XL  [ **BCL2L1** ] , and Bag remained unchanged.  Ref: 12874837 J Cell Biochem, 2003
- We demonstrated that Dox and Taxol induced apoptosis through down regulation of XIAP and phosphorylation of Bcl 2  [ **BCL2** ]  in a concentration dependent manner without changing expression of Bcl xL  [ **BCL2L1** ]  in H460 cells.  Ref: 12883037 Mol Cancer Ther, 2003
- Paraffin embedded sections of 49 ovarian carcinoma were immunostained for survivin, bcl 2  [ **BCL2** ] , bcl x  [ **BCL2L1** ] , bax, and p53.  Ref: 12808063 Mod Pathol, 2003
- It was reported that SCF c kit, Bcl 2  [ **BCL2** ]  and Bcl xl  [ **BCL2L1** ]  inhibited the apoptosis while caspase 3, Fas, Bax and clusterine stimulated it.  Ref: 12861841 Zhonghua Nan Ke Xue, 2003
- Frequency of survivin, bcl 2  [ **BCL2** ] , bcl x  [ **BCL2L1** ] , bax, and p53 was 73.5%, 36.7%, 93.9%, 77.6%, and 60.4%, respectively.  Ref: 12808063 Mod Pathol, 2003
- There was no difference found in the expression of proapoptotic Bax and Bcl X  [ **BCL2L1** ]  s or antiapoptotic Bcl 2  [ **BCL2** ]  and Bcl X  [ **BCL2L1** ]  L genes nor in the expression of the tumor suppressor gene p53.  Ref: 12885947 J Leukoc Biol, 2003
- No change was detected in Bcl 2  [ **BCL2** ] , Bax, or Bcl xL  [ **BCL2L1** ]  proteins.  Ref: 12830043 J Thorac Cardiovasc Surg, 2003
- The apoptotic pathways thought to be employed by epidermal keratinocytes in the various cell layers are reviewed, with special emphasis on the protective mechanisms such as proto oncogenes bcl 2  [ **BCL2** ]  and Bcl XL  [ **BCL2L1** ] , growth factors, and the NFkB pathway in protecting keratinocytes from premature apoptosis during the process of upward migration and differentiation.  Ref: 12884460 J Drugs Dermatol, 2003
- Bcl 2  [ **BCL2** ]  and Bcl X  [ **BCL2L1** ]  expression was assessed semi quantitatively using a score based on the percentage of positive cells and staining intensity.  Ref: 12893071 Cancer Detect Prev, 2003
- Immunoprecipitation studies revealed that Nbk interacts with Bcl x  [ **BCL2L1** ]  L and Bcl 2  [ **BCL2** ]  but not with Bax.  Ref: 12853473 EMBO J< MedlineTA>EMBO J, 2003
